# Investigating the long-term impact of experiencing a major disaster in mid-adulthood on body mass index and waist circumference: A prospective birth cohort study

**DOI:** 10.1016/j.ssmph.2025.101781

**Published:** 2025-03-18

**Authors:** Eggleton Phoebe, Boden Joseph, Harvet Anne, Deng Bingyu, McLeod Geraldine, Campbell Malcolm, Hobbs Matthew

**Affiliations:** a Faculty of Health, University of Canterbury, Christchurch, 8041, Canterbury, New Zealand; b GeoHealth Laboratory, Geospatial Research Institute, University of Canterbury, Christchurch, 8041, Canterbury, New Zealand; c Christchurch Health and Development Study, University of Otago, Christchurch, 8011, Canterbury, New Zealand; d Expertise France, AFD Group, Paris, France; e Department of Geography and Planning, School of Environmental Sciences, University of Liverpool, Liverpool, United Kingdom; f School of Earth and Environment, University of Canterbury, Christchurch, 8041, Canterbury, New Zealand; g College of Health, Wellbeing and Life Sciences, Sheffield Hallam University, Sheffield, Yorkshire, United Kingdom

## Abstract

• Examines association between earthquake exposure and weight-change at age 40 years.
• The associations were adjusted by prior life course covariates.
• Exposure to an earthquake did not increase body mass index at six-years follow-up.
• Exposure to an earthquake increased waist circumference at six-years follow-up.
• A dose-response relationship was evident by severity of exposure for waist circumference.

## 1 Introduction

Despite growing recognition of their importance, longer-term studies examining the impacts of major disasters on health remain scarce, with multiple sources highlighting this significant gap in research (Takahashi et al., 2016, 2020; Zheng et al., 2017). Experiencing adverse or stressful life events during childhood or adulthood can be associated with a higher odd of developing and living with obesity and associated chronic diseases (Palmisano et al., 2016). Globally, the prevalence of individuals living with obesity has almost tripled since 1975 (World Health Organisation, 2021) which is associated with impaired health and quality of life, increased risk of long-term medical complications, and reduced lifespan (Farrell et al., 2021; Pereira et al., 2005; Ross et al., 2016). Of concern, the 2020/21 New Zealand Health Survey (NZHS) found that around 1 in 3 adults (aged 15 years and over) were classified as obese (34.3 %) which was up from 31.2 % in 2019/20 (Ministry of Health, 2022). Moreover, the 2020/21 NZHS found that around 1 in 8 children (aged 2–14 years) were classified as living with obesity (12.7 %), up from 9.5 % in 2019/20 (Ministry of Health, 2022). Prior to this, the rate of obesity among children had been relatively stable. Mirroring international trends (Taxová Braunerová et al., 2021), there are large inequities by ethnicity and deprivation in New Zealand (Bowie et al., 2013; Hobbs et al., 2019). For instance, the prevalence of individuals living with obesity among adults differs by ethnicity, with 71.3 % of Pacific, 50.8 % of Māori, 31.9 % of European/Other, and 18.5 % of Asian adults estimated to be living with obesity in the 2020/21 NZHS (Ministry of Health, 2022). In addition, emulating international evidence, adults and children living in the most socioeconomically deprived areas were 1.6 and 2.5 times as likely to be obese as adults living in the least deprived areas (Ministry of Health, 2022). The causes of obesity and weight gain are complex and multifaceted in aetiology including an interplay of multiple genetic, metabolic, behavioural, and environmental factors (Farrell et al., 2021; Hobbs et al., 2019). Although, it is plausible that sudden life stressors, particularly those linked to climate change and major disasters, may also contribute to increases in the prevalence of obesity and weight gain among populations in disaster-prone areas as demonstrated in supplementary material 1 (Hallegatte, 2014).

Disasters are a state of event, or a situation associated with natural hazards characterised by negative impacts to society in the forms of human and environmental damages (Cadag, 2020). Some research suggests that major disasters are increasing in frequency and severity worldwide due to climatic changes (Emanuel, 2005; Hallegatte, 2014; Hikichi et al., 2019), adversely affecting a greater number of people worldwide. However, other research suggests that the economic losses from disasters have been increasing in recent decades largely attributable to population and economic growth in disaster-prone areas (Botzen et al., 2019). The future impacts of disasters are set to increase (Botzen et al., 2019) which highlights the importance of understanding the impact of disasters on the populations affected by them. There has been growing research into the psychosocial consequences of major disasters including hurricanes, floods, and earthquakes (Devakumar et al., 2023; Fergusson, Horwood, et al., 2014; North & Pfefferbaum, 2013; Ripoll Gallardo et al., 2018). Despite this, less research has examined the impact of disasters on physical health such as changes in body mass index or waist circumference.

Experiencing a major disaster, often classified as a stressful life event, may be associated with weight gain due to the physiological and behavioural responses triggered by such events. However, previous studies on other types of adverse or stressful life events and change in weight are generally cross-sectional and findings are often mixed and depend perhaps on the stressor (Kuijer & Boyce, 2012; Ogden et al., 2009; Proper et al., 2013). Despite this, recent prospective research and some older evidence have shown that adults at a healthy weight may respond differently to adverse and stressful life events than those who were overweight (Proper et al., 2013; Rookus et al., 1988). In addition, proposed biological mechanisms are mostly related to physical stress responses, DNA methylation in genes associated with obesity risk, disturbed emotion regulation, increased appetite and a tendency towards (emotional) overeating (Derks et al., 2021; Hemmingsson, 2018; Kaufman et al., 2018; Kuijer & Boyce, 2012; Michopoulos et al., 2015). Other stress-endorsing factors include a lack of social support relationships which have been associated with a 10 % higher likelihood of an increase in waist circumference when compared to those with high supportive relationships who were significantly less likely to increase body mass index and waist circumference (Kershaw et al., 2014). It is therefore suggested that social support is protective of body mass index and waist circumference (Kouvonen et al., 2011), however social support has not often been considered as an influencing factor in a post-disaster setting. Moreover, from a disaster perspective, recent panel studies following the 2011 Great East Japan earthquake and tsunami showed that residential relocation due to housing destruction was associated with increased body weight and deterioration in physical activity and mental health (Takahashi et al., 2016) as well as increases in body mass index in children (Zheng et al., 2017). In addition, a four-year prospective study of disaster survivors of the 2011 Great East Japan earthquake and tsunami showed an increase in incidence of metabolic syndrome among older survivors who relocated to temporary housing (Takahashi et al., 2020). Relative to evidence on outcomes such as mental health (Norris et al., 2002; North & Pfefferbaum, 2013), fewer studies have robustly explored the link between disaster exposure and changes in body size outcomes, specifically over a longer-term period.

On September 4th, 2010, a large earthquake, measuring 7.1 moment magnitude (M_w_) saw the commencement of the Canterbury Earthquake Sequence (CES) in Canterbury, Aotearoa, New Zealand (Bannister & Gledhill, 2012). The initial earthquake was followed by a severe aftershock on the February 22, 2011 at 12:51 p.m. measuring 6.2 (M_w_) (Kaiser et al., 2012). The effects seen in the aftermath of the disaster disrupted the built environment within the Canterbury region as well as the individuals living within the region (Potter et al., 2015). Within Christchurch, the main city of Canterbury, two multi-storey buildings collapsed, with significant amounts of rubble falling to the streets from surrounding buildings. In the initial 24 h of the disaster 6659 people were injured and 182 killed (Ardagh et al., 2012). Two additional major earthquakes on the June 13, 2011 (6.0 M_w_) and the December 23, 2011 (5.8 M_w_) caused disruption to the recovering region and form the foundation of the CES (Potter et al., 2015).

In a post-disaster setting, it is evident that there are benefits from utilising longitudinal datasets, where multiple observations prior to and following a disaster are available. However, the inherently unpredictable nature of disasters highlights the rarity in obtaining such data to research impacts and recovery post-disaster (Fergusson, Horwood, et al., 2014). Current understanding examining links between exposure to a major disaster and changes in physical health outcomes are restricted by several limitations. First, the majority of evidence is cross-sectional or descriptive in study design (Desjardins et al., 2023; Hobbs & Atlas, 2019; Hobbs et al., 2022). Second, while longitudinal studies exist, there have been few birth-cohort studies based in locations that were subsequently exposed to a major disaster, which means that the evidence base is limited. Third, of the studies that do investigate the association between exposure to a major disaster and body weight or the likelihood of living with obesity, few have standardised and measured body weight outcomes, thus limiting confidence in the robustness of the outcomes. Finally, of the limited studies that are longitudinal, few have robust birth-cohort study designs with high-quality pre-disaster data as well as a long-term follow-up period with most lasting only a few years. A combination of unique circumstances in Christchurch, New Zealand means that we can investigate using a birth-cohort study the association between exposure to a major disaster (e.g. the CES) and measured body mass index and waist circumference obtained in 2017 when cohort members were aged 40 years. We hypothesise that individuals who had the highest severity of exposure to the disaster will have the largest body mass index and waist circumference at age 40 years, six-years following the disaster. Among mechanisms previously identified, we hypothesise that sudden life stressors in the form of experiencing of a major disaster, other stressful life events and the influence of social support networks may contribute to the findings.

## 2 Methods

### 2.1 Study design and participants

Data in this study were collected as part of the Christchurch Health and Development Study (CHDS), a prospective birth-cohort study comprised of 1265 children born in the Christchurch, New Zealand urban region during a 4-month period from April to August 1977. This cohort has been studied on 24 occasions from birth to age 40 years using a combination of interviews with parents and participants, standardised testing, teacher report, and medical record data (Buchanan et al., 2023). All aspects of data collection received ethical approval from Southern Health and Disabilities Ethics Committee, approval number 16_STH_144, and all data were collected with the explicit written consent of study participants.

At ages 30 (in 2007), 35 (in 2012), and 40 years (in 2017), participants completed a regular interview. During the age 35 years (in 2012) interview, in the year following the CES, members of the CHDS cohort were asked to state their exposure to the major earthquake sequence. Within the CHDS cohort, 543 (57.0 %) reported exposure to at least one of the major four earthquakes which comprise the CES. This subgroup then participated in a separate interview based on a series of questions relating to the CES and the aftermath which took approximately 1 h to complete.

### 2.2 Exposure

First, data were obtained on the immediate impacts of the CES. For each of the four major earthquakes, cohort members were asked several questions relating to the severity of the shaking experienced and the immediate consequences of the shaking in terms of damage to furniture, personal possessions; structural damage to their home and other buildings; loss of services and/or damage to infrastructure such as power, phone, water, and sewerage; the extent of liquefaction, flooding, or other land damage; and related issues. The questions asked were derived from the Modified Mercalli Earthquake Intensity Scale which has been outlined in detail previously (Dowrick, 1996; Fergusson, Horwood, et al., 2014). The indices used for the first measure are summarised in Table S1 in online supplementary material 2. Second, data were obtained on the consequences of the CES (Fergusson, Horwood, et al., 2014). Cohort members were questioned about the level of stress and difficulties resulting from earthquake-related disruptions to their personal/family lives and daily activities. Questioning spanned the following domains: housing repairs, accommodation, and insurance issues; disruption of employment/loss of income; changes to daily routines; consequences for their wider family in terms of health, housing, employment, and related issues; and ongoing problems with infrastructure such as drainage, sewerage, and street access. Again, as outlined previously in detail (Fergusson, Horwood, et al., 2014), the questions were modelled on the modified Holmes and Rahe Social Readjustment Rating Scale (Holmes & Rahe, 1967) used to assess life-event exposure. A summarised table representing the second measure is presented in Table S2 in online supplementary material 2. A combination of the first and second measures determined the overall impact of the CES on cohort members. As outlined previously (Fergusson, Horwood, et al., 2014), the measures just described were included in a series of factor models. These analyses showed that when correlated specificity was considered, all items loaded on a common factor that represented the severity of the impact of the earthquakes. An account of the construction of this measure has been provided previously (Fergusson, Horwood, et al., 2014).

Consistent with previous evidence (Fergusson, Horwood, et al., 2014) and for the purposes of data display and the present analyses, the overall measure of earthquake exposure was used to classify the cohort into 5 categories comprising: (1) those who had no exposure to the earthquakes and (2) for those who were exposed, a further 4 groups representing quartiles on the distribution of the total earthquake impact/consequences score with quartile four (Q4) the most severely exposed.

### 2.3 Outcomes

The two key outcomes were body mass index and waist circumference measured at age 40 years. At age 30 years, measures of cohort members height (cm), weight (kg), and waist circumference (cm) were obtained and at age 40 years, measures of weight and waist circumference were again obtained. These measures were taken in cohort members’ homes by trained staff using standardised instruments; Seca 214 portable stadiometers to measure height; Tanita HD-351 scales to measure body weight and a measuring tape to measure waist circumference. Direct assessment was not always possible because some interviews had been conducted with the cohort member on Skype or the telephone. In these cases, the cohort member self-reported their measurements (approximately 20 % of the cohort). Validity of assessing body mass index on the basis of self-report as opposed to measured data was previously examined on a subsample of the cohort at age 30. This showed a correlation of r = 0.96 between assessments of body mass index based on self-report and standardised measurements (Fergusson, McLeod, & Horwood, 2014; McLeod et al., 2016). Using this information, body mass index (kg/m^2^) scores were calculated for respondents. Both body mass index and waist circumference served as outcome variables for this analysis.

### 2.4 Covariates

A range of covariates were selected from the CHDS database to adjust the association between exposure to the CES and body mass index and waist circumference for two reasons: 1) potential confounding factors and 2) selection processes attributable to pre-earthquake factors associated with earthquake exposure. The selected covariates included factors known to be predictive of body mass index and waist circumference outcomes in the CHDS cohort and/or potentially correlated with earthquake exposure, spanning the following domains: prior body mass index and waist circumference at age 30 years; childhood family sociodemographic background and family functioning; exposure to child abuse and family violence; and personal characteristics and family circumstances at the onset of the earthquakes before the cohort members entered the earthquake sequence. The potential correlation between the aforementioned covariates and earthquake exposure have previously been described in detail by authors (Fergusson, Horwood, et al., 2014). Specifically, in this study we were able to control for waist circumference at age 30 years, mothers education at birth, socioeconomic status at birth, parental offending (obtained at age 15 years from parent of cohort member), parental illicit drug use (obtained at age 11 years from parent of cohort member), childhood (<16 years) sexual abuse (obtained at ages 18 and 21 years from cohort member), parental use of physical punishment or childhood (<16 years) physical abuse (obtained at ages 18 and 21 years from cohort member), cohort member educational achievement level (obtained at each assessment from age 18–25 years) and social support at age 35. For further details on covariate descriptions please see online supplementary materials 3.

### 2.5 Statistical analysis

Bivariate associations were tested between a binary classification of exposed or not and then the five-group classification of the extent of earthquake exposure (severity) and the outcomes of body mass index and waist circumference. Significant bivariate associations between earthquake exposure and both body mass index and waist circumference were controlled for potential confounding and selection processes by fitting a series of regression models in which each outcome was modelled as a function of the binary classification of exposed or not and then the five-group measure of earthquake exposure (severity) and the series of covariate factors previously described. From the fully adjusted models, estimates of effect size (unstandardised beta coefficient from linear regression) and corresponding 95 % confidence intervals for a 1-step increase in earthquake exposure (severity) were obtained. Reference categories were always not exposed. STATA V18 (StataCorp, 2023) was used to complete all statistical analyses.

## 3 Results

### 3.1 Descriptive statistics

Table 1 presents the descriptive statistics for exposed or not, and then splits exposure into quartiles from least to most exposed by the measured outcomes of body mass index and waist circumference at age 40 years. Those who were exposed to the CES (n = 543) had a slightly higher body mass index and higher waist circumference than those who were not exposed (n = 409). For instance, mean waist circumference in those exposed was 94.08 cm compared to 90.44 cm in those not exposed and mean body mass index was 28.67 for those exposed compared to 27.80 for those not exposed. More notable differences were seen when body mass index and waist circumference were examined by severity of exposure. The difference was most pronounced in those in the highest severity of exposure for waist circumference (p < 0.002) as opposed to body mass index when examined by quartile of exposure severity (Q4) relative to the least exposed (Q1). For example, those in the most exposed category had a waist circumference of 95.18 cm relative to those in the least severe exposed category (93.13 cm).

**Table 1:** Descriptive statistics of body mass index and waist circumference at age 40 year, stratified by extent of earthquake exposure.

Outcome	Not exposed (n = 409, 43 %)	Exposed (n = 543, 57 %)	Quartile of exposure severity among those exposed				p value^a^
			Q1 (Lowest severity)	Q2	Q3	Q4 (Highest severity)	
**Body Mass Index** (kg/m^2^)	27.80	28.67	27.80	28.72	28.43	28.36	0.04
**Waist circumference** (cm)	90.44	94.08	93.13	93.50	94.52	95.18	0.002

Note: T-test for difference test between those exposed and unexposed. Age 40 years mean body mass index = 28.38; mean waist circumference 92.58 cm.

Results are shown as mean values.

### 3.2 Associations between earthquake exposure, body mass index and waist circumference at age 40 years

We examined the associations between earthquake exposure and earthquake severity and the outcomes of body mass index and waist circumference whilst adjusting for a series of covariates. Table 2 shows the association between earthquake exposure and body mass index at age 40 years. In the unadjusted model, exposure to the earthquake was not related to a higher body mass index at age 40 years (b = 0.87 [95 % Confidence Interval (CI) −0.16, 1.91]) relative to those not exposed which was attenuated further in adjusted models (b = 0.38 [95 % CI -0.20, 0.95]). In addition, no association was seen when examined by severity of exposure and body mass index at age 40 years. Table 3 provides the results of the models examining the association between earthquake exposure and waist circumference at 40 years. In the unadjusted model, exposure to the earthquake was associated with an increase in waist circumference at age 40 years (b = 3.64 [95 % CI 1.63, 5.66]) relative to those cohort members not exposed. This association was slightly attenuated in the fully adjusted model (b = 2.11 [0.38, 3.84]). Finally, when examined by severity, more severe earthquake exposure was related to a higher waist circumference at age 40 years. In the unadjusted models, relative to those not exposed those in Quartile 2 (Q2) (b = 3.06 [95 % CI -0.01, 6.12], Q3 (b = 4.08 [95 % CI 1.06, 7.11]) and Q4 (b = 4.74 [95 % CI 1.69, 7.80] was related to higher waist circumference. In the fully adjusted models, the association remained for Q2 and Q4 but was fully attenuated for Q1 and Q3

**Table 2:** Examining the association between exposure (binary) and severity (categorical) of exposure the earthquake and body mass index (kg/m^2^) at age 40 years.

	Unadjusted model	Fully adjusted
**Earthquake exposure** Not exposed Exposed	Reference 0.87 [-0.16, 1.91]	Reference 0.38 [-0.20, 0.95]
**Earthquake severity** 0 (not exposed) 1 2 3 4	Reference 0.93 [-0.61, 2.46] 0.64 [-0.82, 2.10] 0.56 [-0.86, 1.98] 1.36 [-0.06, 2.78]	Reference 0.76 [-0.08, 1.59] 0.43 [-0.37, 1.22] 0.05 [-0.72, 0.83] 0.35 [-0.43, 1.12]

Note: Models were adjusted for: body mass index at age 30 years, mother's education at birth, socioeconomic status at birth, parental offending (missing data imputed), parental illicit drug use (missing data imputed), exposure to sexual abuse in childhood, parental use of physical punishment (childhood physical abuse), educational achievement level, social support at age 35.

Results are shown as beta coefficient [95 % CI].

**Table 3:** Examining the association between exposure (binary) to the earthquake and severity (categorical) and waist circumference (cm) at age 40 years.

	Unadjusted model	Fully adjusted
**Earthquake exposure** Not exposed Exposed	Reference **3.64 [1.63, 5.66]∗**	Reference **2.11 [0.38, 3.84]∗**
**Earthquake severity** 0 (not exposed) 1 2 3 4	Reference 2.69 [-0.33, 5.72] 3.06 [-0.01, 6.12] **4.08 [1.06, 7.11]∗** **4.74 [1.69, 7.80]∗**	Reference 1.71 [-0.78, 4.21] **2.84 [0.46, 5.23]∗** 1.28 [-1.04, 3.61] **2.55 [0.24, 4.86]∗**

Note: Models were adjusted for: waist circumference at age 30 years, mother's education at birth, socioeconomic status at birth, parental offending (missing data imputed), parental illicit drug use (missing data imputed), exposure to sexual abuse in childhood, parental use of physical punishment (childhood physical abuse), educational achievement level, social support at age 35. Bold denotes p < 0.05∗.

Results are shown as beta coefficient [95 % CI].

## 4 Discussion

This study examined the impact of exposure to a major disaster, the CES, on objectively measured height and weight used to calculate body mass index as well as measured waist circumference. The nature of the study proposed two groups where 57 % of a well-studied birth-cohort was living in an area subject to a major disaster and 43 % were living outside of this area or were unexposed. Our prospective cohort study contributed two key findings to the literature. First, there was an association between earthquake exposure and increased waist circumference at age 40 years six-years following the major disaster. Second, there was some evidence of a dose-response association when examined by severity with those cohort members most severely impacted associated with the largest increases in waist circumference.

The major findings add evidential rigour to existing evidence through using a prospective birth-cohort study design. After controlling for a range of confounding factors, exposure to a major earthquake sequence was associated with increased waist circumference but not body mass index at six-years post-disaster at age 40 years. Importantly, the study design permitted control of confounding and offered a comprehensive measure of earthquake exposure that also accounted for the adverse personal, social, and economic circumstances that resulted from the earthquakes. Specifically, at six-years follow up those exposed to the earthquake were estimated to have a 2 cm greater waist circumference than those not exposed and those most severely exposed had a 2.5 cm higher waist circumference than those not exposed. In our paper, we aim to correlate the statistical significance of a 2.5 cm increase in waist circumference with its clinical relevance. There is notable scarcity in the literature regarding the clinical significance of increases or decreases in waist circumference. For instance, Cerhan et al. (2014) modelled their association using “clinically intuitive” 5 cm increments. Additionally, Ross et al. (2020) highlighted the importance of a significant decrease in waist circumference by at least 5 cm. Finally, in an expert analysis from the American College of Cardiology, it was argued that even modest reductions in waist circumference could be clinically meaningful (Jha et al., 2021). This underscores the need for further data and a consensus on specific thresholds for waist circumference increases or reductions that could be considered clinically significant. Furthermore, our findings support those of an important meta-analysis examining the impacts of earthquake exposure on several adverse later-life health outcomes including mortality, cardiovascular diseases, mental health, and problems related to lifestyle (Ripoll Gallardo et al., 2018). The meta-analysis mainly found a modest increase in glycated haemoglobin levels from 2 to 12 months after earthquakes, however, they did not find an increase in body mass index (Ripoll Gallardo et al., 2018). This supports our findings of no association with body mass index however, the meta-analysis only considered body mass index, and our study adds to evidence by also considering how earthquake exposure is related to measured waist circumference.

In addition to body mass index, our study perhaps highlights the importance of including waist circumference as a valuable anthropometric measure which has already been highlighted as critical for patient management, obesity diagnosis and cardiometabolic disorders screening (Green, 2016). Indeed, the monitoring of obesity using body mass index alone may underestimate the prevalence of obesity (Gearon et al., 2018) and other meta-analyses indicate that we should take into account other factors such as ethnicity when considering body mass index and waist circumference to assess health outcomes (Seo et al., 2017). Moreover, this meta-analysis concluded that waist circumference is better than body mass index in predicting diabetes (Seo et al., 2017). As New Zealand is a multi-ethnic nation with already known different health outcomes such as for diabetes, high blood pressure and obesity, it is perhaps worth considering waist circumference, as well as, or rather than body mass index in future analyses of health outcomes in post-earthquake studies. Finally, the International Atherosclerosis Society and International Chair on Cardiometabolic Risk Working Group on Visceral Obesity recommend in a Consensus Statement that waist circumference should be routinely measured in clinical practice. Routinely measured waist circumference can provide independent information for morbidity and mortality prediction (Ross et al., 2020).

Our study supports some previous evidence which has shown that exposure to a disaster is related to adiposity. For instance, following the 2011 Great East Japan Earthquake and tsunami residential relocation due to housing destruction was associated with increased body weight (Nagao et al., 2024). The study showed that evacuation was associated with the risk of becoming overweight seven years after the earthquake (Nagao et al., 2024). Other research has shown a potential association between early life exposure to tsunami and civil war in Sri Lanka with increased body mass index and higher body fat (Devakumar et al., 2023). This also supports other evidence which has shown changes in dietary patterns post-disaster. After the 2011 Japan earthquake dietary changes have been observed between 2011 and 2013 (Ma et al., 2021). It appeared that evacuees had lower typical patterns and higher juice patterns in comparison with non-evacuees (Ma et al., 2021). Additionally, another cohort study of evacuees showed that people living in evacuation shelters were more likely to have lower consumption of fruits and vegetables, and dairy products (Zhang et al., 2017). Our findings support these previous results, and this should be considered when responding to disasters for healthcare providers and policymakers. Furthermore, alongside social determinants of health, previous exposition to disasters can trigger adverse health outcomes in the long term. This exposition could be another determinant of health risk – modifying factor in the development of obesity for people in affected regions. It is therefore crucially important to study and understand health outcomes after disasters to increase preparedness and health system responses with targeted interventions at the forefront. Longer-term monitoring of populations who experience a major disaster would aim to prevent adverse health effects including obesity or weight change, mental health issues and health inequity. Monitoring systems should be integrated through post-disaster health plans, with resource allocation considered for ongoing health support.

We respond to calls to use prospectively collected data in geospatial research (Desjardins et al., 2023; Hobbs & Atlas, 2019). Our findings extend previous literature which have often focused on mental health demonstrating that among the consequences of exposure to a major disaster are increased risks for major depression, anxiety disorders, and post-traumatic stress disorder (Fergusson, Horwood, et al., 2014). Further, our study includes social support as a covariate, which is an association that has not been included in post-disaster research to date. A strength of our research is the availability of information which predates the occurrence of the disaster and as such our study design was, therefore, able to effectively address the problem of recall bias in most studies conducted in post-disaster settings (Hikichi et al., 2019). However, the birth-cohort study design also has several limitations. First, cohort members who stay in Christchurch are often of lower socioeconomic status and may have more mental health issues whereas people who moved are generally of higher socioeconomic status a phenomenon widely recognised now as the healthy migrant effect in wider literature (Biddle et al., 2023; Deng, Campbell, et al., 2024; Deng, McLeod, et al., 2024; Hobbs et al., 2023). In addition, our findings were also limited to populations in their mid-30s and while our design is robust it is plausible that associations may differ for different age groups, by disaster and by country of location. It is possible that the impacts of the earthquakes varied with age, with the result that effects on younger and older populations may be different from those found for this cohort. We also cannot state exactly where an individual was at the time the disaster struck (Campbell et al., 2021), however, we have a better understanding through more recent research (Eggleton et al., 2024). Despite this our time frame for assessment, which was several years after the onset of the earthquake sequence, is able to delineate the longer-term effects of earthquake exposure which are important when examining factors such as body mass index and waist circumference which may take time to change unlike other more proximal outcomes such as mental health or health behaviours such as alcohol consumption or smoking behaviours. In addition, we were unable to account for important health behaviours such as dietary quality or change over time. Therefore, future research should explore mediation effects to explore the mechanism by which any association between earthquake exposure and adverse outcomes for body composition may operate. A study composed by Shiba and colleagues (2020) could be used as a model for future studies, where investigating the effect that relocation has on a population exposed to disaster could provide an interesting perspective. Moreover, the study did not account for cohort members who were not in the region at the time of the CES, although had close family members who were impacted. An analysis of the potential impact on this group within the cohort could provide a separate examination in the future.

## 5 Conclusion

In summary, we present findings from one of the few studies internationally to examine the long-term effects of an earthquake on body mass index as well as waist circumference using a prospective birth-cohort study design. A key strength is the study design and the ability to control for a range of covariates prior to the event taking place which is to the authors knowledge one of the first studies to do so internationally. Nevertheless, we report rigorous evidence on the impact of a major earthquake on two body composition measures post-disaster which have important implications for longer-term physical health outcomes. Our findings suggest that experiencing a major disaster may be related to adverse consequences for physical health in terms of waist circumference. As a result, it is essential to implement targeted interventions and establish monitoring systems for populations exposed to a major disaster.

## CRediT authorship contribution statement

**Eggleton Phoebe:** Writing – review & editing, Writing – original draft, Methodology, Formal analysis, Conceptualization. **Boden Joseph:** Writing – review & editing, Supervision, Data curation, Conceptualization. **Harvet Anne:** Writing – review & editing, Writing – original draft, Conceptualization. **Deng Bingyu:** Writing – review & editing, Data curation. **McLeod Geraldine:** Data curation, Conceptualization. **Campbell Malcolm:** Writing – review & editing, Supervision. **Hobbs Matthew:** Writing – review & editing, Writing – original draft, Validation, Supervision, Resources, Project administration, Methodology, Investigation, Funding acquisition, Formal analysis, Conceptualization.

## Ethical statement

All aspects of data collection received ethical approval from Southern Health and Disabilities Ethics Committee, approval number 16_STH_144, and all data were collected with the explicit written consent of study participants.

## Funding

This project was funded by a New Zealand Health Research Council Emerging Researcher First Grant (22/528). The Christchurch Health and Development Study age 40 assessment was supported by a 10.13039/501100001505Health Research Council of New Zealand Programme Grant [16/600]. Previous work has been supported by the National Child Health Research Foundation (10.13039/501100001515Cure Kids), the 10.13039/501100001530Canterbury Medical Research Foundation and the 10.13039/501100009940New Zealand Lottery Grants Board.

## Declaration of competing interest

The authors declare the following financial interests/personal relationships which may be considered as potential competing interests: Matthew Hobbs reports financial support was provided by New Zealand Health Research Council Emerging Researchers First Grant. If there are other authors, they declare that they have no known competing financial interests or personal relationships that could have appeared to influence the work reported in this paper.

## Data Availability

The authors do not have permission to share data.
