# Supplementary material for: Investigating the long-term impact of experiencing a major disaster in mid-adulthood on body mass index and waist circumference: A prospective birth cohort study
Source: SSM Popul Health. 2025 Mar 18;30:101781. doi: 10.1016/j.ssmph.2025.101781 (PMC11984572; doi:10.1016/j.ssmph.2025.101781)
Supplement: Multimedia component 1 [file mmc1.docx]

# Online supplementary materials

**Supplement 1:** Conceptual Framework

**Physical health outcomes**

(body mass index, waist circumference)

**Disaster exposure**

(Canterbury earthquake sequence (2010-11))

**Environmental impact**

(severity of damage)

**Cardiometabolic health**

(e.g. increased stress, altered metabolism)

**Direct exposure**

(immediate experience)

**Indirect exposure**

(community-level disruption)

**Health impacts**

(physiological, mental)

**Supplement 2:** Summary of the indices and domains used to assess exposure to the CES

S1. Indices Used to Determine the Immediate Impact of the Canterbury Earthquake Sequence on Christchurch Health and Development Study Cohort Members

| The 11 indices |
| --- |
| 1. Severity of shaking experienced during earthquakes. |
| 1. Extent to which small objects rattled, toppled or fell off shelves. |
| 1. Extent to which cupboard doors were thrown open and contents ejected. |
| 1. Extent to which small items of furniture, appliances (e.g. TV, computer), or light machinery slid of toppled over. |
| 1. Extent to which large fixtures, appliances (e.g. fridge, filing cabinet) or heavy machinery slid or toppled over. |
| 1. Extent of damage to buildings where participant was located at the time of the earthquake. |
| 1. Extent of loss of services (power, phone, water, etc) where participant was located at the time. |
| 1. Extent of damage to household effects. |
| 1. Extent of damage of home. |
| 1. Extent of loss of services (power, phone, water, etc) to home. |
| 1. Extent of land damage (liquefaction, flooding, subsidence, etc) in the area around participant’s home. |

(Fergusson et al., 2014)

S2. Domains Used as a Measure of the Consequences That Christchurch Health and Development Study Cohort Members Experienced During the Canterbury Earthquake Sequence

| Five domains |
| --- |
| 1. Housing repairs, accommodation and insurance issues. |
| 1. Disruption of employment/loss of income. |
| 1. Disruption to daily routines. |
| 1. Consequences for wider family members in terms of health, housing, employment and related issues. |
| 1. Ongoing problems with disruption of services/infrastructure repairs (e.g. drainage, sewerage, street access). |

(Fergusson et al., 2014)

**Supplement 3:** Further details on the CHDS covariates

Selected measures included the following: maternal age in years at the time of the cohort member’s birth; maternal education at the time of the cohort member’s birth assessed on a 3-point scale (no formal qualifications; high school qualifications; tertiary technical qualifications/university degree); family socio-economic status at the time of the cohort member’s birth assessed using the 6-level Elley-Irving Scale of Socio-economic Status for New Zealand (Elley & Irving, 1976), with higher scores implying lower SES; parental history of adjustment problems including criminal offending and illicit drug use. Parental criminal offending (missing data imputed) was defined at age 15 years where the young person's parents were asked if they had a record for criminal offending. On the basis of responses to this questioning 12.7% of the sample were classified as having a parental history of offending. Parental illicit drug use (missing data imputed) was defined when sample members were aged 11 and their parents were questioned about parental usage of illicit drugs including cannabis. On the basis of this questioning 27.5% of the sample were classified as having parents who used cannabis or other illicit drugs.

Childhood Sexual Abuse was defined at ages 18 and 21 years where sample members were questioned about their experience of sexual abuse during childhood (<16 years) (Fergusson et al., 1996). Questioning spanned an array of abusive experiences from episodes involving non-contact abuse (e.g. indecent exposure) to episodes involving attempted or completed intercourse. Sample members who reported an abusive episode were then questioned further about the nature and context of the abuse. Using this information a 4-level scale was devised reflecting the most extreme form of sexual abuse reported by the young person at either age. This classification was: no sexual abuse; non-contact abuse only; contact sexual abuse not involving attempted or completed intercourse; attempted/completed oral, anal, or vaginal intercourse. Parental use of physical punishment (childhood physical abuse) was defined at ages 18 and 21 where sample members were asked to describe the extent to which their parents used physical punishment during childhood (Fergusson & Lynskey, 1997). Separate questioning was conducted for mothers and fathers. This information was used to create a 4-level scale reflecting the most severe form of physical punishment reported for either parent: parents never used physical punishment; parents rarely used physical punishment; at least one parent used physical punishment on a regular basis; at least one parent used physical punishment too often or too severely or treated the respondent in a harsh or abusive manner.

Educational achievement level was defined at each assessment from age 18-25 years where cohort members were questioned in detail about their educational history since the previous assessment, including attainment of high school qualifications, enrolment in tertiary education and degree attainment. In the New Zealand education system, students attend high school for up to 5 years (Year 9-Year 13). The minimum school leaving age is 16. Most students turn 16 in Year 11; however, the majority of students remain in high school at least until the end of Year 12. The high school qualifications framework that applied for this cohort included the following qualifications. At the end of Year 11 students were eligible (but not required) to take School Certificate examinations. Most students sat examinations in 4-6 subjects. Student performance in each subject was graded from A to E, with a C representing a “pass” grade in the subject. In Year 12 (6th form) students could complete an approved course of study (usually 5 or 6 courses in various subjects) leading to a qualification known as Sixth Form Certificate. Similarly, in Year 13 (7th form) students could complete a qualification known as Higher School Certificate: this qualification was awarded to students who completed 5 years of high school education from Year 9 and who completed at least three subjects above Year 12 (6th form) level. Finally, in Year 13 students intending to progress on to university could sit University Bursary examinations. Those who attained a sufficient grade percentage in these examinations were eligible for entry into university, and those who attained at a higher level again were awarded a bursary to support their university study. The University system of qualifications is similar to overseas systems of qualifications, and requires the equivalent of three years full-time study to achieve a Bachelor’s degree, a further one to two years for Honors or a Masters degree, and the equivalent of three years for a Doctoral degree. For the purposes of the present analysis the information on educational achievement was used to classify individuals on a 7 level scale reflecting the highest level of educational attainment by age 25 years. This scale was: 1 = gained no high school qualifications; 2 = obtained at least one pass grade in one School Certificate subject; 3 = attained Sixth Form Certificate; 4 = attained Higher School Certificate; 5 = qualified for a University Bursary; 6 = enrolled in university; 7 = completed a bachelors or higher level degree qualification (M = 4.22, SD = 2.24).

Finally, social support was assessed at ages 18, 21, 25, 30, 35 and 40 (Buchanan, 2023). At age 35 assessment, participants were asked to identify the number of people that would provide various types of support (none, one, 2-3, 4-5, 6+). Scores were summed providing an estimate of the number of friends as different types of support.

## References

Buchanan, M. (2023). *Protective factors following cumulative childhood adversity* [University of Otago].

Elley, W. B., & Irving, J. C. (1976). Revised socio-economic index for New Zealand. *New Zealand Journal of Educational Studies*, *11*(1), 25–36.

Fergusson, D. M., & Lynskey, M. T. (1997). Physical punishment/maltreatment during childhood and adjustment in young adulthood. *Child Abuse & Neglect*, *21*(7), 617–630. <https://doi.org/10.1016/S0145-2134(97)00021-5>

Fergusson, D. M., Lynskey, M. T., & Horwood, L. J. (1996). Childhood Sexual Abuse and Psychiatric Disorder in Young Adulthood: I. Prevalence of Sexual Abuse and Factors Associated with Sexual Abuse. *Journal of the American Academy of Child & Adolescent Psychiatry*, *35*(10), 1355–1364. <https://doi.org/10.1097/00004583-199610000-00023>

Fergusson, D. M., Horwood, L. J., Boden, J. M., & Mulder, R. T. (2014). Impact of a major disaster on the mental health of a well-studied cohort. *JAMA Psychiatry*, *71*(9), 1025–1031.
